# Seven decades of southern range dynamics of Canada lynx

**DOI:** 10.1002/ece3.7364

**Published:** 2021-03-09

**Authors:** Robby R. Marrotte, Jeff Bowman

**Affiliations:** ^1^ Environmental & Life Sciences Graduate Program Trent University Peterborough ON Canada; ^2^ Ontario Ministry of Natural Resources & Forestry Wildlife Research & Monitoring Section Trent University Peterborough ON Canada

**Keywords:** Canada lynx, Great Lakes region, harvest records, *Lynx canadensis*, range dynamics, spatiotemporal

## Abstract

The range of the Canada lynx (*Lynx canadensis*) has contracted substantially from its historical range. Using harvest records, we found that the southern range of the lynx in Ontario in the late 1940s collapsed and then, in a short period of time, increased to its largest extent in the mid‐1960s when the lynx range spread south of the boreal forest for a decade. After this expansion, the southern range contracted northwards beginning in the 1970s. Most recently, there has been a slight expansion between 2010 and 2017. We have attributed these dynamics on the southern range periphery to the fluctuation of the boreal lynx population in the core of the species' range. In addition, connectivity to boreal lynx populations and snow depth seemed to condition whether the lynx expanded into an area. However, we did not find any evidence to suggest that these changes were due to anthropogenic landscape disturbances or competition. The boreal lynx population does not reach the peak abundance it once did, without which we would not expect to see large expansions of the southern lynx range as in the mid‐1960s. Our results suggest that the southern lynx range in Ontario has been driven by the magnitude of the boreal lynx population cycle, connectivity to the boreal forest, and snow conditions. Future persistence of lynx in the southern range periphery will likely depend on dynamics in the range core.

## Graphical abstract

We used Canada lynx harvest records to understand how its southern range outside of the boreal forest in Ontario has changed over the past 7 decades. We found that the southern lynx range was predominantly driven by the population dynamics of lynx in the boreal forest and areas with deep winter snow were often found within the southern lynx range. We did not find support for the anthropogenic effect of roads and competition from bobcats and coyotes.

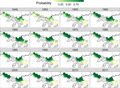

## 1 INTRODUCTION

Over the past century, the range of many species has changed, often attributed to climate change and land cover modification (Laliberte & Ripple, 2004; Thomas, 2010; Walther et al. 2002). A species can respond to environmental changes by exploiting resources at the extremities of its niche (Sexton et al. 2017), by phenotypic plasticity (Nicotra et al. 2010; Valladares et al. 2014) or by adaptation (Williams et al. 2008). However, the rate at which current conditions are changing might make adaptation impossible for many species because the process of natural selection is too slow (Davis & Shaw, 2001). Consequently, species will have to track their bioclimatic niche (Visser, 2008) and their ability to do so will influence their persistence (Bell & Gonzalez, 2011; Schloss et al. 2012; Travis et al. 2013).

Terrestrial species that have ranges near the poles will be limited in their ability to track climate because they are limited by the availability of space to move to higher latitudes (Kerr & Packer, 1998). Therefore, many unique cold adapted species will eventually perish unless they have sufficient phenotypic plasticity or somehow adapt to warmer conditions and to new biotic interactions. Understanding how and why the warmer range edge of cold adapted species has been changing would help us in making better informed conservation decisions, since anthropogenic change is not slowing down.

The Canada lynx (*Lynx canadensis*) is an iconic carnivore that largely resides in the boreal forest of North America and its northern range edge has some expansion potential into taiga landscapes but is generally bounded by tundra and the Arctic Ocean (Poole, 2003). The lynx is a habitat specialist because it almost exclusively preys on snowshoe hares (*Lepus americanus*) in the boreal forest (O’Donoghue et al. 1998). Consequently, its population dynamics are highly coupled to the 8‐ to 11‐year population cycle of the snowshoe hare, mirroring it with a 1‐ to 2‐year delay (Poole, 2003). Since presettlement times, the Canada lynx range in North America has contracted by 40% (Laliberte & Ripple, 2004). However, most of this range reduction took place prior to the 20th century and was attributed to unregulated harvest and habitat loss due to land clearing during European colonization (Hoving et al. 2004; McKelvey, 2000; Poole, 2003; de Vos, 1964; Vos & Matel, 1952).

Canada lynx are predominantly found in areas where snowshoe hare density is above 0.5 per hectare (Berg et al. 2012; Hodges et al. 2009; Ivan et al., 2014; Ruggiero et al. 2000; Zahratka & Shenk, 2008). In the southern periphery of the lynx range, hare population densities have declined compared with historic levels (Aubry et al., 2000; Hodges, 2000; Murray, 2000) and this most likely accounts for the contraction of the lynx from its historic range (Poole, 2003). Following years with high peak hare abundance, Canada lynx appear to migrate from range core to range periphery as a result of density‐dependent dispersal (McKelvey, 2000). Such dispersal pulses might lead to higher occupancy of the southern range periphery following periods of high hare abundance (King et al. 2020; McKelvey, 2000; Murray et al. 2008). Consequently, lower peaks in hare abundance might decrease the likelihood of dispersal of lynx into the southern periphery (Licht et al. 2019; Poole, 2003). Southern dispersal might also be limited in some locations by habitat quality and connectivity with the range core (Buskirk, 2000; Holbrook et al. 2019; Ruggiero et al. 2000; Walpole et al. 2012).

The warming climate might also affect the lynx indirectly through its main food source the snowshoe hare. The timing between molt and season change for the snowshoe hare is important in decreasing predation rates (Zimova et al. 2016). A changing snow regime could increase snowshoe hare predation rates by increasing the rate of mismatch between snowshoe hare molt and season change. Increased predation rates might also reduce the amplitude of the hare cycle (Krebs, 2010).

Climate change will also open formerly inhospitable habitat to new species in the lynx range. Bobcats (*Lynx rufus*) and coyotes (*Canis latrans*) are two species whose ranges are expanding into the geographic range of lynx, often at the same time as the lynx range is contracting (Hody & Kays, 2018; Marrotte et al. 2020). Both species have smaller feet than Canada lynx; consequently, they might not be able to support as much weight as lynx in deep snow without sinking (Parker et al. 1983; but see Kolbe et al. 2007). This might be one factor that has hindered the bobcat from invading Canada lynx territory in the past (Marston, 1942; McCord, 1974; Murray et al. 2008; Parker et al. 1983). However, since the climate is warming, and snow depths across the southern periphery of the lynx range are shallower, southern competitors might be less hindered by snow, increasing their competitive potential (Buskirk, 2000; Peers et al. 2020; Ruediger et al. 2000; Scully et al. 2018). In fact, Parker et al. (1983) found that after several years of low snow the bobcat invaded the lowlands of Cape Breton while the Canada lynx left the area. Marrotte, Bowman, and Wilson (2020) found that deep winter snow in the Great Lakes region limited bobcat expansion northward, suggesting that greater expansion will result from additional climate warming. Recently, Peers et al. (2020) found that while snow depth has decreased across their study area in the boreal forest, snowshoe hare survival decreased while predation by coyotes increased in areas with shallow snow.

The lynx once occurred in 24 of the United States (McKelvey, 2000) and currently occurs in 7 (US Fish & Wildlife Service, 2017). The lynx is designated as “threatened” in the contiguous United States (US Fish & Wildlife Service, 2000), although its protection status is being debated and it might be removed from the list of endangered species in the United States (Cummings et al. 2020). In Canada, the lynx occupies 95% of its historic range (Poole, 2003). However, it is designated as provincially endangered in Nova Scotia (Parker, 2001), and New Brunswick (New Brunswick Endangered Species Regulation, 2013) and was extirpated from Prince Edward Island (Poole, 2003). Further analysis has demonstrated that the range of lynx in British Columbia has been stable since the 1930s (Gooliaff & Hodges, 2018). In contrast, the lynx range in eastern Ontario appears to have contracted northwards by 175 km from 1972 to 2010 (Koen et al. 2014).

We estimated the past extent of the Canada lynx southern range in Ontario, Canada, using harvest records and then determined whether the spatial–temporal patterns could be attributed to snowshoe hare and boreal lynx population dynamics, connectivity, climate, land use, and competition. We predicted that years with low Canada lynx abundance in the boreal forest were associated with a reduction in the extent of the southern lynx range. We also predicted that areas with high human disturbance, shallow snow, presence of competitors, and with low connectivity to boreal lynx populations were less likely to be occupied by lynx as part of the southern range.

## 2 MATERIAL AND METHODS

### 2.1 Study area

**FIGURE 1:**
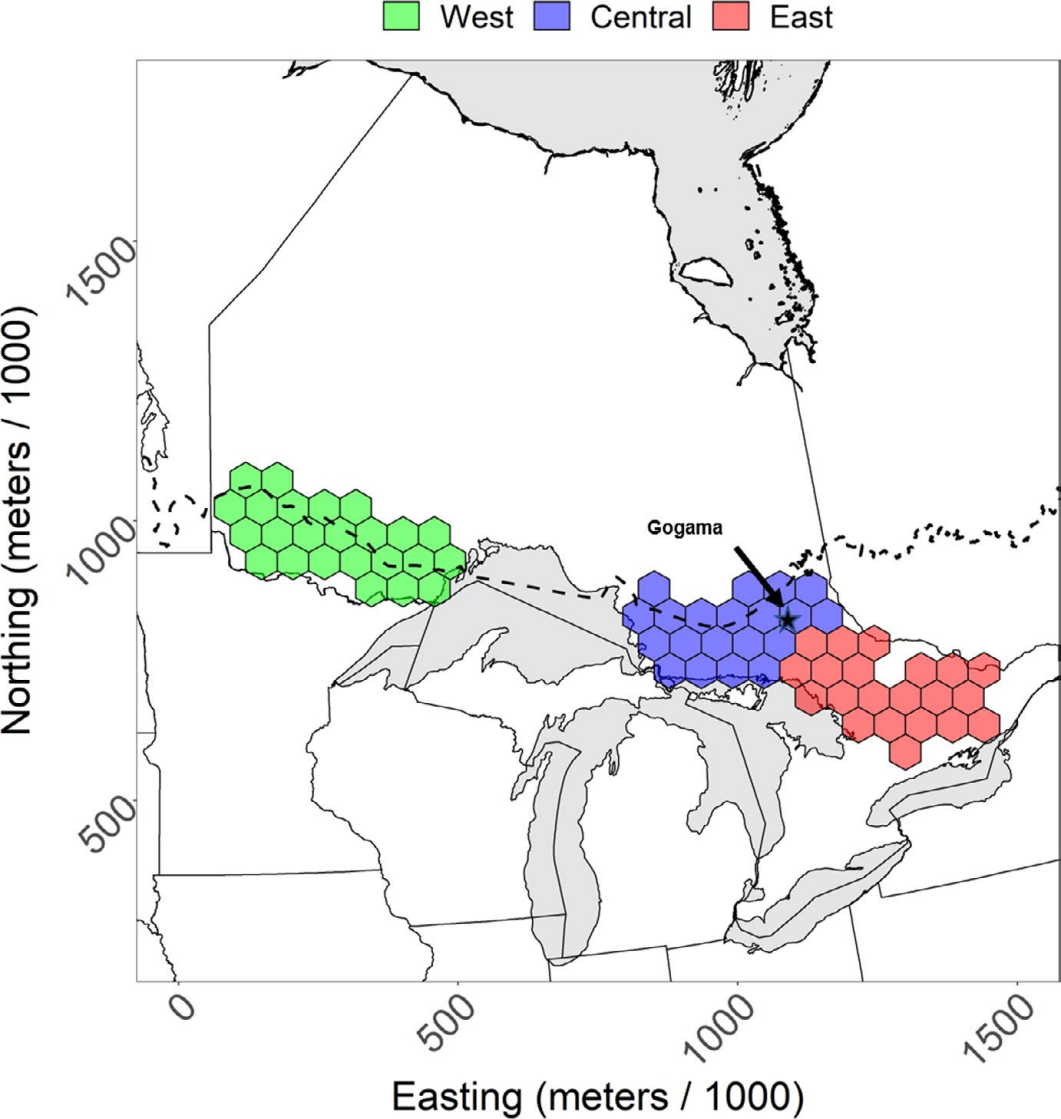
Sampling units in the southern Canada lynx range in Ontario, Canada, used to estimate the probability of harvesting a lynx between 1948 and 2017. The dashed black line is the southern limit of the boreal forest by Brandt (2009). Spatial layers for administrative boundaries were gathered from the Database of Global Administrative Areas (https://gadm.org/)

Our study area was the southern periphery of the lynx range in Ontario, Canada, which we defined as an area between the southern margin of the boreal forest and areas south of the boreal forest where lynx occurred at least once between 1948 and 2017 in Ontario, Canada (Figure 1). To first identify the boreal forest, we used the spatial layer supplied by Natural Resources Canada that was derived from maps from the early 1970s to the late 2000s (Brandt, 2009). We then defined our study area as the region where lynx have occurred south of the boreal forest. We added to the study area an additional band of forest that extended 1 sampling unit (defined below) or 65 km north of the southern boundary of the boreal forest to account for uncertainty in both the boreal limit and the uncertainty in our harvest records. There were 3 distinct southern range zones in Ontario. The west and central zones were separated by Lake Superior and were also within 100 km of the boreal forest, whereas the east zone was farther away. We used these zones to illustrate regional trends in range change, since these zones had different spatial and temporal patterns.

The southern lynx range periphery was predominantly found in the Great Lakes‐St Lawrence Forest, which is a transition zone between the boreal and deciduous forest (Boucher et al. 2009). The Great Lakes‐St Lawrence forest is dominated by white pine (*Pinus strobus*), red pine (*Pinus resinosa*), hemlock (*Tsuga canadensis*), American beech (*Fagus grandifolia*), yellow birch (*Betula alleghaniensis*), and sugar maple (*Acer saccharum*) (Rowe, 1972).

### 2.2 Harvest records

Long‐term spatial data on terrestrial species are quite rare. Fortunately, wildlife agencies track furbearer harvest each year. Such records contain important information that can be used to monitor and study the change in range, spatial distribution, and population dynamics of several species that are harvested for their fur (Hayne, 1949; Viljugrein et al. 2001).

Ecologists have used fur harvest data to address fundamental questions in ecology (Bulmer, 1974; Elton & Nicholson, 1942; Keith, 1963; Krebs et al. 1995). There are, however, some issues with using fur returns. Trapping effort should be accounted for (DeVink et al. 2011; Dorendorf et al. 2016), and generally, the location of capture is only available at a coarse geographic level.

**FIGURE 2:**
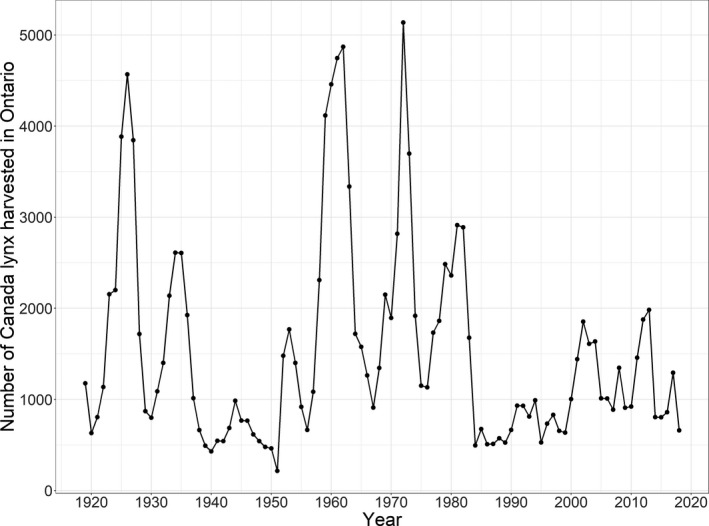
Number of Canada lynx harvested in Ontario, Canada, between 1919 and 2018. Values earlier than 1947 were from Novak (1987a, 1987b). Later values were aggregated from the Ontario fur returns that were used in this study

The Ontario Ministry of Natural Resources and Forestry has been compiling furbearer trapping records since the beginning of the 20th century (Figure 2). A registered trapline system in Ontario began in the late 1940s, and therefore, spatially referenced annual harvest records are available beginning in 1947. Trapping of furbearers in Ontario takes place within a township or on a registered trapline. Traplines are designated as areas on public land where trappers are licensed to harvest furbearers. Hereinafter, we refer to townships and traplines as trapping units. We georeferenced these records using the appropriate trapping unit map for each harvest record.

### 2.3 Spatial and temporal coverage

Boundaries of trapping units changed occasionally due to regulation changes. Therefore, we divided our study area into 146 equal‐sized hexagons or sampling units of 2,731 km^2^ each. The area of these hexagons was based on the largest trapping unit found in the study area between 1947 and 2017. We assigned each trapping unit to the hexagon that its centroid fell into. All the information in each trapping record was then aggregated to the hexagonal sampling unit for each year. There were years where records were completely missing for all sampling units (1969, 1970, 1975, 1986, 1989, and 1991), years where many records were missing (1947, 1972, and 1992) and other years where certain sampling units had the occasional missing record. Consequently, temporal coverage of sampling units varied from 65 years to only 4 years for the 71‐year period between 1947 and 2017.

Due to the variability of spatial and temporal coverage, we restricted our analysis to sampling units that had good temporal coverage. We first restricted our analysis between 1948 and 2017, because the trapline system was not fully implemented in 1947 and therefore had limited spatial coverage. We further restricted our analysis to sampling units that had at least one lynx that was harvested from 1948 to 2017. Next, we omitted sampling units that had >5 years of consecutive missing data or more than 10 years of missing records. This left 82 of the previous 146 sampling units. Finally, we removed sampling units that contained on average less than 1,000 km^2^ of trapping unit surface area between 1948 and 2017. These sampling units were all found either near the periphery of large water bodies, near political boundaries, or near an area that had trapping restrictions (Provincial Parks or crown game preserves). This left a final sample size of 65 sampling units.

### 2.4 Estimating the spatial and temporal range

We used the R package “mgcv” to fit Hierarchical Generalized Additive Models (HGAM) to estimate the probability of harvesting a Canada lynx within sampling units across space and time (Woods, 2011). Hierarchical Generalized Additive Models allow the modeling of nonlinear relationships between covariates and responses where the shape of the function varies between grouping levels (Pedersen et al., 2019). We used multiple time series of data that were clustered into different groups (i.e., different sample units). The HGAM framework allowed us to appropriately deal with the spatial, temporal, and spatiotemporal autocorrelation in model residuals. Other methods (e.g., Generalized Additive or Linear Models) would not allow us to simultaneously account for nonlinear spatial and temporal variation.

Our general approach was to compare variation in harvest probability across years and across sampling units to assess effects of predictor variables. We first built several models that combined our effort predictors. After comparing several alternatives, we used thin plate smoothers for each predictor, because we expected nonlinear relationships. We also compared two different spatial–temporal tensor product smoothers (Augustin et al. 2013; Eickenscheidt et al., 2018; Wood et al. 2013; Zhou et al. 2019). In each spatial–temporal structure, we modeled the yearly temporal variability with a cubic regression smoother. The spatial structure was modeled with a spatial discrete process using a Markov Random Field (MRF) or a thin plate (TP) smoother on the spatial coordinates.

We used Relative Maximum Likelihood to fit our models. We set the number of knots “k” to 5 for each effort predictor, to 65 for all spatial smoothers, and to 40 for the year smoother. We set the spatial and temporal knots to high values based on our highest computational capabilities. However, the “gam” function in the mgcv package will fit models using penalized likelihood to estimate parameters for each basis function, therefore increasing the number of knots simply makes computation longer and does not overfit the model. Some basic functions may be penalized to the point where their estimates are zero in the final model fit (Pedersen et al., 2019).

We then estimated the range of the Canada lynx across space and time by predicting the probability of trapping a lynx with an average value of effort. We identified the areas that had at least a 50% chance of harvesting a Canada lynx for each year between 1948 and 2017.

### 2.5 Trapping effort covariates

We investigated 3 types of effort measures: trapping area or frequency, harvest, and market‐based measures. Our trapping area or frequency‐based measures were the total number of trapping units and the area covered by trapping units within each sampling unit of each year. Our first harvest‐based effort measure was the total number furbearers harvested. We also thought that the density of American marten (*Martes americana*) harvested on a trapline would be a good measure of trapping effort. Martens are sympatric with lynx and are regularly one of Ontario's most sought‐after furbearers. Marten fur is valuable and harvest rates are relatively consistent, which would help to discriminate between trappers that are active and those that are not. Consequently, we employed marten harvest as a second index of trapper effort (Fryxell et al. 2001; Webb et al. 2008). The price of lynx fur is also an important factor that can govern harvest patterns of lynx (DeVink et al. 2011; Dorendorf et al. 2016). Our market‐based measure was the average lynx pelt price from the previous year.

For all animal‐based measures of effort, we investigated the log of the absolute number, density, and the average number of animals across trapping units, since the number of animals trapped varied exponentially between trapping units. In total, we had 9 effort predictors, but we did not investigate models that combined total furbearer harvest and American marten harvest, since these measures were not independent. We also only investigated models that included the total number of trapping units, the area occupied by those trapping units, and the average pelt price. Consequently, we compared 6 different effort models to find the best model to account for effort bias in harvesting a lynx.

We calculated the yearly average price of lynx pelts that originated from Ontario using the fur‐return summaries from a variety of sources. We gathered summaries collected by Statistics Canada (http://www5.statcan.gc.ca; CANSIM Table 003–0013). The time series ranged from 1970 to 2011, but most of the data from 2010 to 2017 were missing. Therefore, we used summaries provided by the Fur Institute of Canada for 2010–2017 (www.fur.ca). We then added data from 1948 to 1970 provided by Novak (1987a, 1987b).

We corrected for inflation using the Consumer Price Index (CPI) for the province of Ontario also available on the Statistics Canada website (statcan.gc.ca; CANSIM Table 326–0021). For each year, we multiplied the average pelt price by the 2019 CPI and divided these values by the CPI of their appropriate year. This adjusted the average pelt prices to 2019 Canadian dollars. In our analysis, we used the adjusted average pelt price of the previous year for the current year of observation. We assumed that trappers observing a high pelt price were more likely to harvest a lynx in the following year.

### 2.6 Testing hypotheses of range change

We were interested in understanding how the area of the southern range fluctuated over space and time in accordance with different hypotheses. To simplify our analyses, we broke up our subsequent analyses into both spatial and temporal tests.

To test spatial hypotheses, we summed the number of times each sampling unit was part of the lynx range between 1948 and 2017. We then compared these values with each spatial predictor while we controlled for the influence of all other predictors with a partial Spearman rank correlation. We used a nonparametric correlation coefficient, because the response variable and all the covariates were not normally distributed. To test our temporal hypotheses, we calculated the area occupied by the southern lynx range each year and compared each temporal predictor with this time series. We investigated temporal lags of up to 2 years. Temporal stationarity is an important assumption for the association metric to be valid; therefore, we calculated the between year differences for all time series (Priestley, 1988). We then estimated associations with a Pearson correlation coefficient. We resampled without replacement our observations 9,999 times to calculate *p*‐values. We then adjusted our *p*‐values to account for multiple tests using a Bonferroni correction.

### 2.7 Spatial and temporal predictors

We calculated the distance to boreal forest by summing the straight‐line distance between the edge of each sampling unit and the closest boreal forest as defined by our map layer. We used this coarse method because it was simple and repeatable. For human disturbance, we used the major roads in the Ontario Road Network layer as a proxy variable (LIO; geohub.lio.gov.on.ca). For each sampling unit, we calculated the distance to the nearest road in kilometers.

We estimated a snowshoe hare time series by gathering hare abundance data from the Ontario Ministry of Natural Resources and Forestry (OMNRF Unpublished). Monitoring of hare populations is undertaken in the fall (October) through an array of pellet count plots in several locations across the province (e.g., Bendell & Young, 1995). We used pellet data from the longest running snowshoe hare population monitoring (since 1986) in Gogama, Ontario (Figure 1). These data originated from many plots that we aggregated to a single measure that indicates the average number of hare pellets. These are the only long‐term data for snowshoe hare population dynamics in the boreal forest of Ontario, and the number of pellets should indicate the density of hares in the surrounding area (Krebs et al. 2001).

We built the boreal forest lynx population time series by gathering all trapping records located within the boreal forest and summed these by year. We wanted our boreal lynx population index to be independent from our response data; therefore, we removed all records used to estimate the lynx range that were outside of the boreal forest (i.e., all records within our hexagonal study areas). We also log‐transformed these boreal lynx harvest values to correct for harvest bias (Royama, 2012).

We created a snow map and time series from weekly measurements gathered from the SNOW network for Ontario wildlife database (OMNRF, 2020; Warren et al. 1998). For each year, we calculated the SDI (Snow Depth Index), which is the sum of all weekly measurements collected at a station over the winter months. We interpolated the data across our study area using ordinary kriging using the “automap” package in R (Hiemstra & Hiemstra, 2013). We then calculated the average SDI for each sampling unit for our spatial map and we calculated the average annual SDI for each year between 1952 and 2017. We removed stations that had less than 16 measurements during the year. This is equivalent to 4 months of winter and captured some early spring and late fall snow events.

We built maps of the occurrence of competitors and their associated time series by counting the number of times each species (bobcat and coyote) was present in the harvest records of each sampling unit over time and space. For our spatial map, we summed the number of years that a competitor was found in each sampling unit. For our time series, we summed the number of sampling units that each species was present in during each year.

We performed all spatial processing and modeling in R version 5.5.1 (R Core Team, 2014). All spatial layers were projected to MNRF Lambert conformal conic (EPSG:3161).

## 3 RESULTS

### 3.1 Quantifying effort

The model that could best account for the effort of harvesting a Canada lynx and the spatial–temporal process in the southern range periphery of Ontario, Canada, included the log‐transformed total number of furbearers harvested and a thin plate smoother on the spatial coordinates (Table S1). This model was 20.914 AIC units lower than all other models and its AIC_w_ was 1.000. The spatiotemporal effort model had an adjusted *R* ^2^ of 0.586 and a deviance explained of 54.1%. Other than the total number of furbearers harvested, the 3 other effort related predictors followed linear relationships (Figure S1). The additive effect of the number of trapping units, the total area, and the average price were not as important as the total number of animals harvested. The probability of harvesting a lynx decreased with the total area harvested while the 3 other predictors had a positive relationship. Also, the influence of lynx pelt price was weak compared with the other predictors.

### 3.2 Range dynamics

**FIGURE 3:**
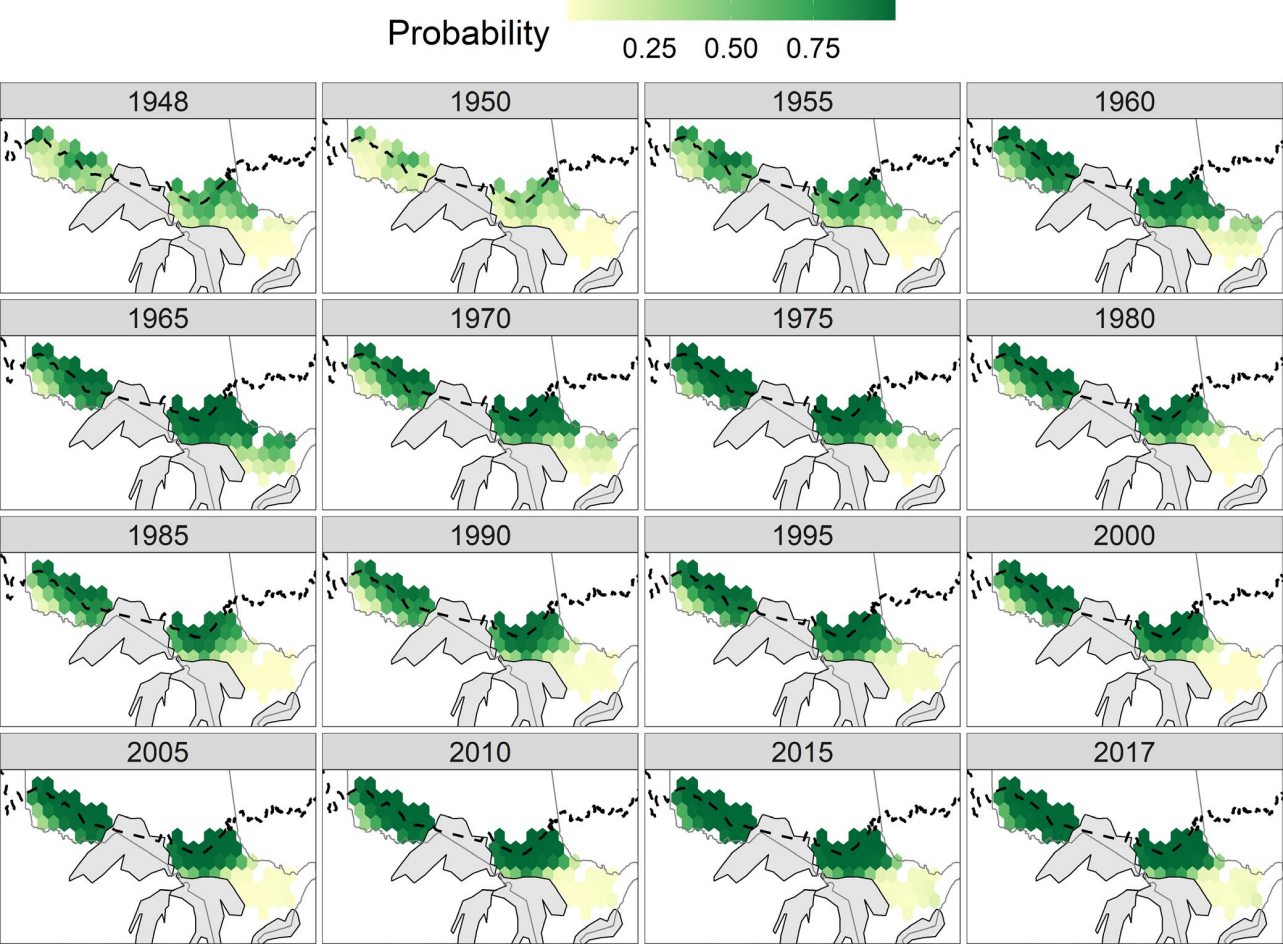
Spatial–temporal pattern of the probability of harvesting a Canada lynx from 1948 to 2017 in Ontario, Canada. The dashed black line is the boreal forest southern limit by Brandt (2009). Spatial layers for administrative boundaries were gathered from the Database of Global Administrative Areas (https://gadm.org/)

The probability of harvesting a Canada lynx south of the boreal forest across Ontario changed through time (Figure 3). During the late 1940s and the early 1950s, the likelihood of harvesting a lynx was at its lowest. However, in the mid‐1960s the probability of harvesting a lynx peaked across the southern range and even trapping units found in the east had a high probability. After this peak lynx period, it then became less likely to harvest a lynx in the east and this pattern continued to 2017. The odds of harvesting a lynx peaked in both the west and central zones in the early 1960s and again in the mid‐1970s, then declined until the 2000s and increased slowly until 2017 to an overall probability of harvest higher than in previous years.

**FIGURE 4:**
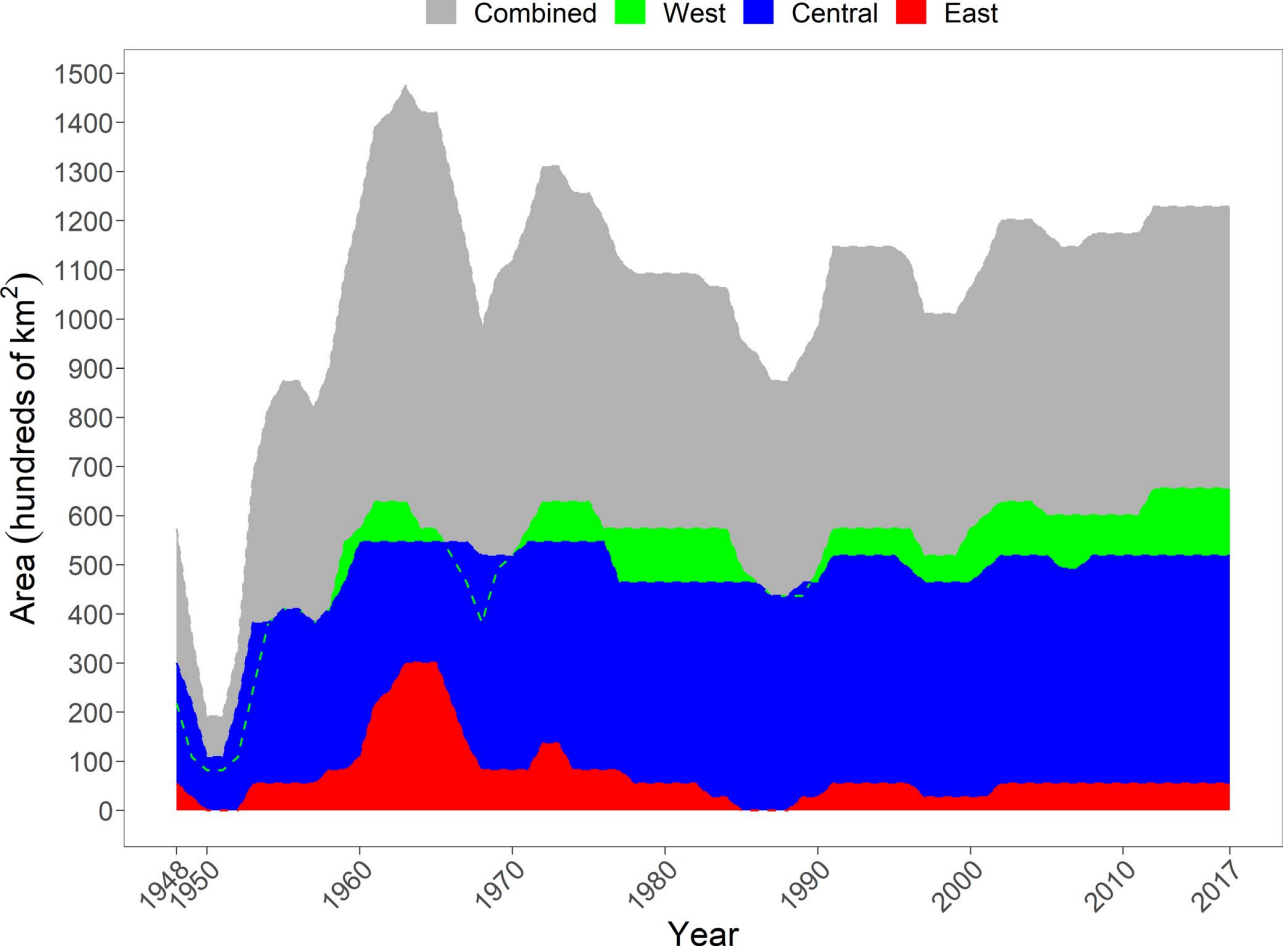
Area of the Canada lynx southern range in Ontario, Canada, between 1948 and 2017. The maximum area of the west, central, and east zone was 65,548, 54,623, and 30,043 km^2^. The area was calculated by summing the area of the sampling units (hexagons) that had a probability of harvesting a lynx over 0.5

To get a better idea of the range dynamics, we calculated the occupied area of the southern range of each zone for each year (Figure 4). In 1950, the total area of the southern lynx range was at its lowest and occupied a total area of 19,118 km^2^. The area of the range peaked between 1963 and 1964 and occupied a maximum area of 147,483 km^2^. This was an area 7.7× larger than during the crash in the late 1940s. From 1970 onwards, the southern range varied much less in size compared with previous years. It declined between 1970 and the late 1980s, but gradually increased until 2017 to a size comparable to the early 1970s. There were also a few notable decreases in range in the periods 1965–1972, 1983–1992, and 1995–2002.

In general, all 3 zones (west, central, and east) followed similar patterns. However, from 1957 to 1964 the east zone increased from 5,462 to 30,043 km^2^, which was a sixfold increase and occupied most of Lanark and Renfrew Counties just west of Ottawa (Figure 3). This increase was not as dramatic in the west and central zones, where there was only a 1.5‐ and 1.3‐fold increase. Although these two northernmost ranges were already closer to their maximum extent of 65,548 and 54,623 km^2^, consequently they could not have increased as intensely during this period. A smaller range contraction in this same Lanark and Renfrew County area also occurred in 1971 to 1973.

From the late 1950s to 2017, the west and central zones varied by 24,581 [40,968–65,548] and 16,387 km^2^ [38,236–54,623]. The west zone reached its maximum area more recently in 2013 and 2017, whereas the central zone reached its maximum area multiple times in the periods 1960–1967 and 1970–1976. The east zone varied quite differently. It increased dramatically twice in the period 1959–1973 and never reached these levels again. After this point, the range varied between 0 and 8,193 km^2^.

We calculated the number of years each sampling unit was within the lynx southern range. Sampling units in the south were less frequently part of the Canada lynx range (Figure S2). In the east zone, sampling units were only part of the range on average 8.7 years over the 70‐year time period. In contrast, sampling units in the west and central zones were part of the range 48.2 and 58.1 years, respectively.

### 3.3 Causes

We predicted that undisturbed areas with deep snow, an absence of competitors, and close proximity to the boreal forest were more likely to be part of the southern range. We found that 2 of 5 of these relationships met our initial expectations (Table 1). Sampling units that were more frequently found within the Canada lynx range were closer to the boreal forest and had deeper average annual snow. We also predicted that years with large numbers of hare and lynx in the boreal forest, low number of competitors, and deep snow increased the extent of the southern Canada lynx range. We found that only 1 relationship met our initial expectations (Table 1); when the number of Canada lynx harvested in the boreal forest increased, the area of southern range increased the following year.

**TABLE 1:** Spatial and temporal relationships

Covariates	Expected relationship	Spatial		Temporal			
		Partial *ρ*	Probability	*r*	Lag	Probability	Span
Distance boreal forest	−	−**0.336**	0.008				
Distance nearest road	+	0.189	0.144				
Average annual SDI	+	**0.454**	0.000	0.129	0	1.000	1952–2017
Bobcat presence	−	0.036	0.783	−0.228	0	1.000	1948–2017
Coyote presence	−	−0.071	0.591	−0.292	0	0.402	1948–2017
Average hare pellets	+			0.337	1	1.000	1986–2017
Boreal lynx harvested	+			**0.504**	1	0.003	1948–2017

Values in bold face are significant. Two‐tailed *p*‐values were calculated from 9,999 permutations. Spatial relationships are partial correlations. Temporal *p*‐values were adjusted with a Bonferroni correction. We only reported the lags that had the absolute highest coefficient. However, all other lags had an adjusted *p*‐value > 0.05. All partial correlation coefficients are Spearman's rank correlation coefficient and temporal correlations are Pearson's correlation coefficients.

## 4 DISCUSSION

We predicted that years with low Canada lynx abundance in the boreal forest were associated with a reduction in the extent of the southern lynx range. We found support for this idea, as it appeared that southern range dynamics were mostly driven by dynamics at the core of the lynx range in the boreal forest. The probability of harvesting a lynx at the southern periphery was positively associated with lynx harvest the year earlier in the boreal forest. Our findings underscore the importance of population dynamics in the core of the lynx range, density‐dependent dispersal, and connectivity with the range core in the boreal forest for the persistence of lynx populations at the southern edge of their range.

We also predicted that areas with high human disturbance, shallow snow, presence of competitors, and with low connectivity to boreal lynx populations were less likely to be occupied as part of the southern range. However, it was not clear due to variation in our models whether anthropogenic effects of roads and our indices of competition from bobcats and coyotes influenced the southern range occupancy of the Canada lynx in Ontario. We did find that areas that had deep winter snow were often found within the southern lynx range. However, this relationship did not vary temporally with the area of the southern lynx range (Table 1). We used this snow depth predictor as an index of climate change, since we thought that the highest impact of climate warming on lynx would be related to the timing of molt of its main prey the snowshoe hare. We also thought that competition would arise in areas with less snow over time and would become more hospitable to coyotes and bobcats. We found that the average annual snow depth was not associated with the temporal dynamics of southern range occupancy by lynx in Ontario, but deep snow was associated with the occurrence of lynx.

There was a weak signal for the temporal dynamics of the coyote, but we did not have enough power to detect a significant relationship given the number of tests we performed (Table 1). It is quite reasonable to think that coyotes are competitors because they are generally found across the southern range apart from a few areas within the boreal forest in Ontario (Figure S3). Predation by coyotes on snowshoe hares might be a mechanism for competition, as has been demonstrated in the Yukon (Peers et al. 2020). Bobcats on the other hand occupied a very small area and generally occurred in the south of the west and central zones (Figure S4). The restricted distribution of the bobcat indicates that the species is not responsible for the range contraction in the east zone, since it is rarely found there. Recent finer scale studies suggested segregation between lynx and bobcats (Marrotte et al. 2020; Morin et al. 2020).

We observed that the southern range of the lynx has recovered from a dramatic decline in the late 1940s (Figure 4). It has never returned however, to the short‐lived maxima we observed during the 1960s. More recently, during 2012–2017, the lynx range has reached its maximum extent in the west and central zones. After the mid‐1980s, the southern range varied less and from 2010 to 2017, seemed to be increasing. Consequently, we did not find the substantial range loss in more recent times that has been observed in parts of the contiguous United States (Ruediger et al. 2000), and in a previous analysis in eastern Ontario (Koen et al. 2014). The stable and somewhat increasing range in Ontario is not unique across the lynx range, since lynx are increasing in numbers in Maine, USA (Simons‐Legaard et al., 2016) and the lynx range in British Columbia has been stable since 1935 (Gooliaff & Hodges, 2018). It is important to remember however that the Canada lynx range across all of North America has contracted substantially from its precolonization extent (Laliberte & Ripple, 2004).

The extensive contraction in the early part of the time series may have extended from 1938 to 1951 (Figure 2). In fact, de Vos and Matel (1952) noted that lynx occurrences were rare at this time and the range was also gradually shrinking. They attributed this decline to ecological changes and overharvesting. The decline prompted the closing of lynx trapping during the 1951–1952 season and a quota system for lynx was established and trapping was reopened the next year (de Vos & Matel, 1952). At the same time, in all of Canada, harvest dropped from 33,054 pelts in 1925 to only 3,734 lynx pelts in 1949 (de Vos & Matel, 1952). Lynx fur returns for each jurisdiction in Canada were an order of magnitude lower during the population crash. In approximately the same period, lynx occurrences and harvest in Wisconsin, Minnesota, and Michigan also dropped (McKelvey, 2000).

Immediately after this large continent‐wide population crash and subsequent range contraction, the southern range in Ontario expanded almost eightfold (Figure 4). The ranged peaked in 1963–1964 and lynx were being harvested more than 100 km south of the boreal forest in southern Ontario for almost 10 years (Figure 3). At the same time, there was an increase in fur returns and occurrences of lynx immediately south in the Great Lakes states (McKelvey, 2000). Similar range expansion and population increase were also present in Alberta, British Columbia, Saskatchewan, Manitoba, and Quebec during this period (McKelvey, 2000; Todd, 1985).

These earlier large fluctuations of the southern lynx range in Ontario and harvest in the Great Lakes states were likely driven by immigration of lynx from the boreal forest (Licht et al. 2019; McKelvey, 2000; Murray et al. 2008; Steury & Murray, 2004). We did see this pattern in our analysis; the southern lynx range changed with the population dynamics of the boreal lynx and this influence decayed away from the core range in the boreal forest (Table 1). Density‐dependent dispersal from the boreal forest likely drives the southern lynx range in the northern Great Lakes region. Consequently, many southern populations are likely maintained by emigration from sources in the range core (Murray et al. 2008; Steury & Murray, 2004). During peak years, individuals venture south and colonize marginal habitat outside of the boreal forest in Ontario and eventually reach the northern Great Lakes states (Mech, 1973, 1980). In more recent times in Ontario, the boreal lynx cycle has not reached the amplitudes it once did (e.g., in the 1960s; Figure S5); therefore, lynx populations at the southern range periphery are less likely to be rescued.

There was a period of slow range contraction from 1970 to the late 1990s, where lynx appeared and quickly disappeared from their southern Ontario range. This period is not unique to Ontario, most jurisdictions followed the same pattern (McKelvey, 2000). In an earlier study, Koen et al. (2014) noted that the largest range loss happened in this period, but we did not see a continuous decline after 1991 as they did. In fact, the range expanded, and the west and central zones were at their largest possible extent and occupied a combined area that was previously unforeseen (Figure 4). Our results probably differ because we were able to assess a longer time series (1972–2010 vs. 1948–2017) and we examined a much larger area.

## 5 CONCLUSION

The southern range of Canada lynx in Ontario is strongly driven by boreal lynx population dynamics, which means that the persistence of lynx populations in what we currently think of as the southern range periphery likely depends on density‐dependent dispersal and connectivity with the range core. Years of high lynx abundance and high rates of dispersal will be required to rescue southern lynx populations. Efforts to understand processes affecting the amplitude of lynx‐hare cycles in the core of the boreal forest and changing the quality of lynx habitat at the southern range edge will both contribute to conserving southern edge populations of this iconic species.

## CONFLICT OF INTEREST

None declared.

## AUTHOR CONTRIBUTIONS

**Robby R. Marrotte:** Conceptualization (lead); data curation (lead); formal analysis (lead); investigation (lead); methodology (lead); project administration (lead); visualization (lead); writing–original draft (lead); writing–review and editing (lead). **Jeff Bowman:** Conceptualization (supporting); formal analysis (supporting); funding acquisition (lead); investigation (supporting); methodology (supporting); project administration (supporting); resources (supporting); supervision (lead); validation (equal); visualization (supporting); writing–original draft (supporting); writing–review and editing (lead).

## Supporting information

Supplementary Material

## Data Availability

The data that support the findings of this study are openly available on Dryad at https://doi.org/10.5061/dryad.f4qrfj6vc.
