# Supplementary material for: Seven decades of southern range dynamics of Canada lynx
Source: Ecol Evol. 2021 Mar 9;11(9):4644–55. doi: 10.1002/ece3.7364 (PMC8093747; doi:10.1002/ece3.7364)
Supplement: Supplementary file 1 — Supplementary Material [file ECE3-11-4644-s001.docx]

# Supplemental Material

Table S1. Summaries of models used to explain the probability of harvesting a lynx while accounting for harvest effort across sampling units in the southern lynx range between 1948-2017 in Ontario, Canada. Covariates unrelated to animals harvested were also included in each of these models. These were the total number of trapping units, the area occupied by those trapping units and the average pelt price. The spatiotemporal pattern was modelled with a tensor product smoother. We modelled the year of harvest with a cubic regression smoother and the spatial process was modelled with a thin plate smoother on the geographic coordinates or with a Markov random field smoother using the neighborhood structure. Harvest-based effort covariates were ln transformed. MRF is Markov Random Field and TP is Thin Plate smoother.

| **Rank** | **Harvest-based Covariate*** | **Spatial Smoother** | **Relative Maximum Likelihood** | **Proportion Deviance Explained** | **R^2^** | **AIC** | **ΔAIC** | **ω** |
| --- | --- | --- | --- | --- | --- | --- | --- | --- |
| 1 | Total Harvest | TP | 1521.568 | 0.541 | 0.586 | 2838.874 | 0.000 | 1.000 |
| 2 | Average Total Harvest | TP | 1533.745 | 0.538 | 0.583 | 2859.789 | 20.914 | 0.000 |
| 3 | Total harvested Density | TP | 1543.371 | 0.536 | 0.582 | 2872.364 | 33.490 | 0.000 |
| 4 | Marten Harvest Density | TP | 1549.976 | 0.535 | 0.582 | 2883.528 | 44.654 | 0.000 |
| 5 | Marten Harvest | TP | 1555.515 | 0.534 | 0.580 | 2894.338 | 55.464 | 0.000 |
| 6 | Marten Average Harvest | TP | 1557.371 | 0.533 | 0.579 | 2896.628 | 57.754 | 0.000 |
| 7 | Total Harvest | MRF | 1611.840 | 0.583 | 0.605 | 3010.789 | 171.915 | 0.000 |
| 8 | Average Total Harvest | MRF | 1618.995 | 0.584 | 0.606 | 3018.095 | 179.220 | 0.000 |
| 9 | Total harvested Density | MRF | 1624.088 | 0.585 | 0.606 | 3022.048 | 183.174 | 0.000 |
| 10 | Marten Harvest Density | MRF | 1616.483 | 0.582 | 0.605 | 3026.693 | 187.819 | 0.000 |
| 11 | Marten Average Harvest | MRF | 1620.269 | 0.583 | 0.606 | 3032.354 | 193.480 | 0.000 |
| 12 | Marten Harvest | MRF | 1620.067 | 0.582 | 0.605 | 3032.467 | 193.593 | 0.000 |


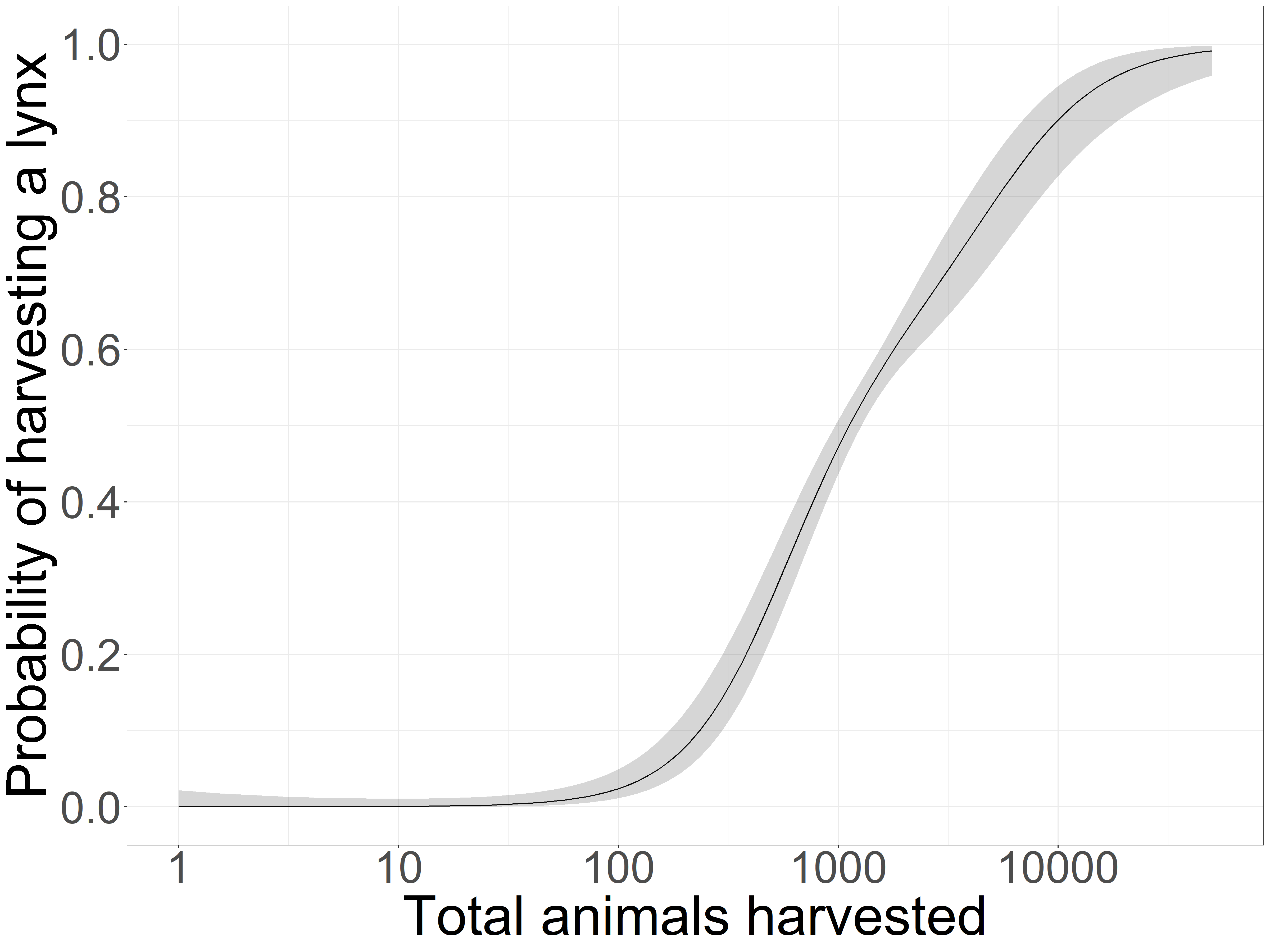

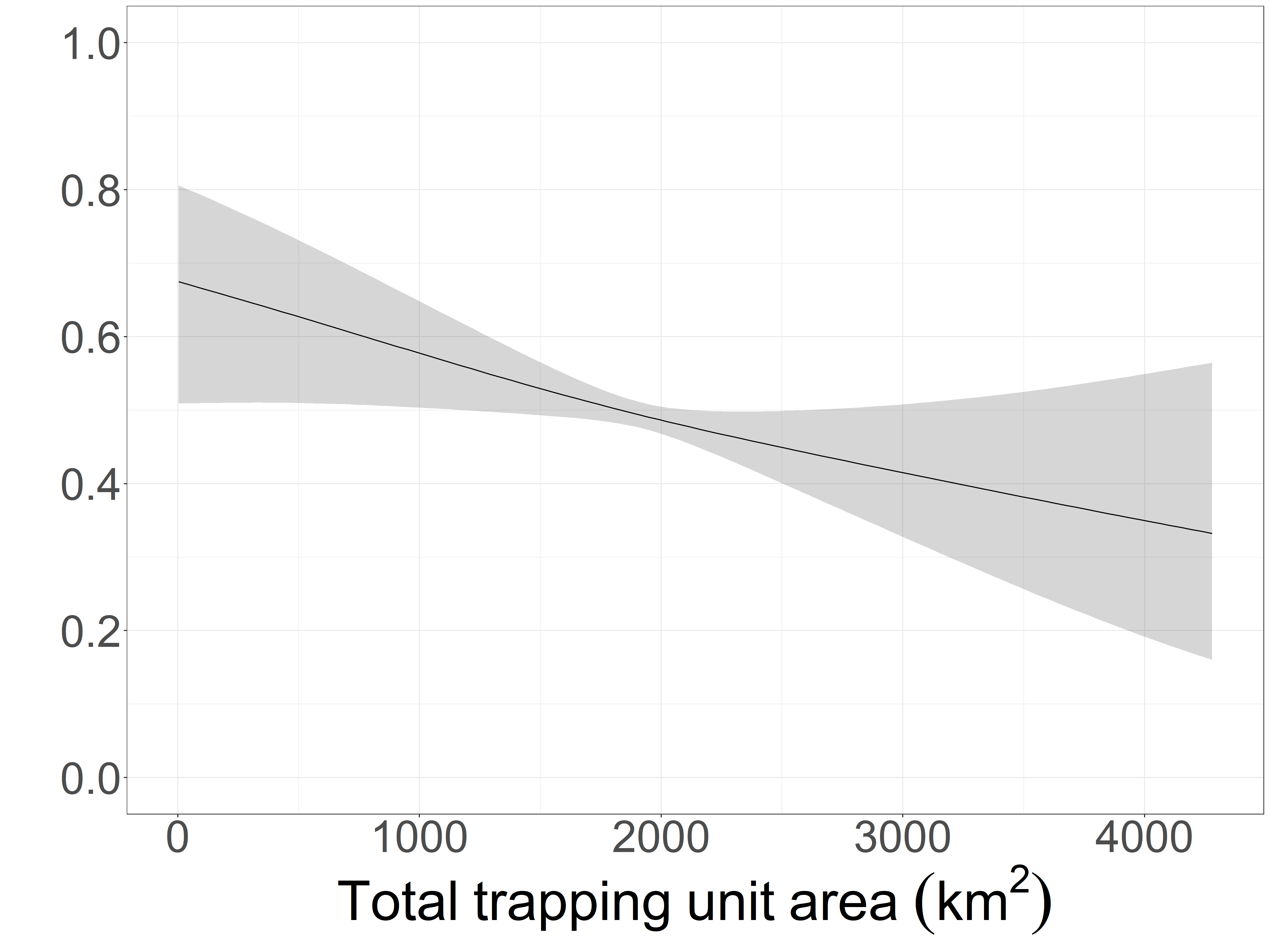

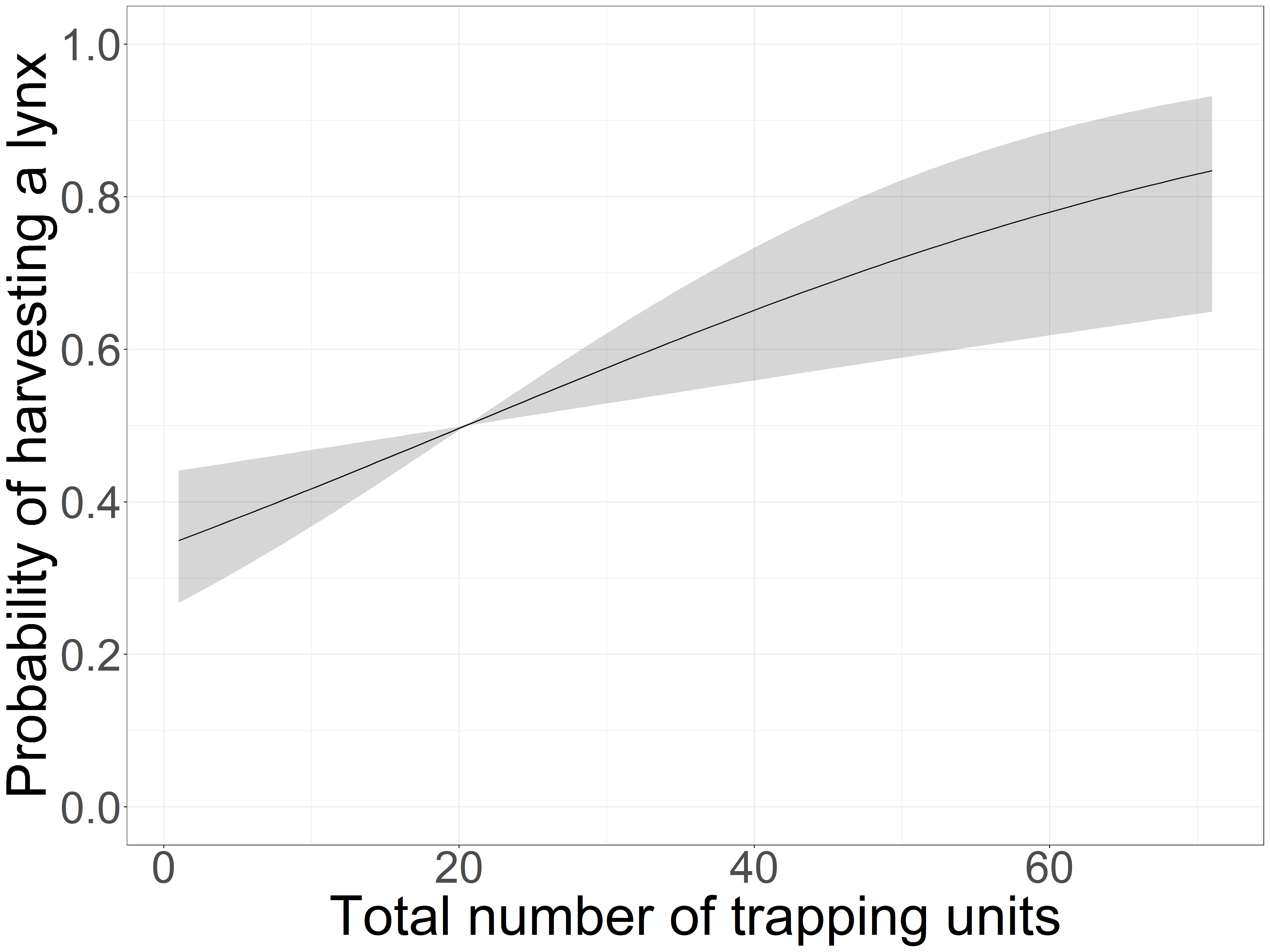

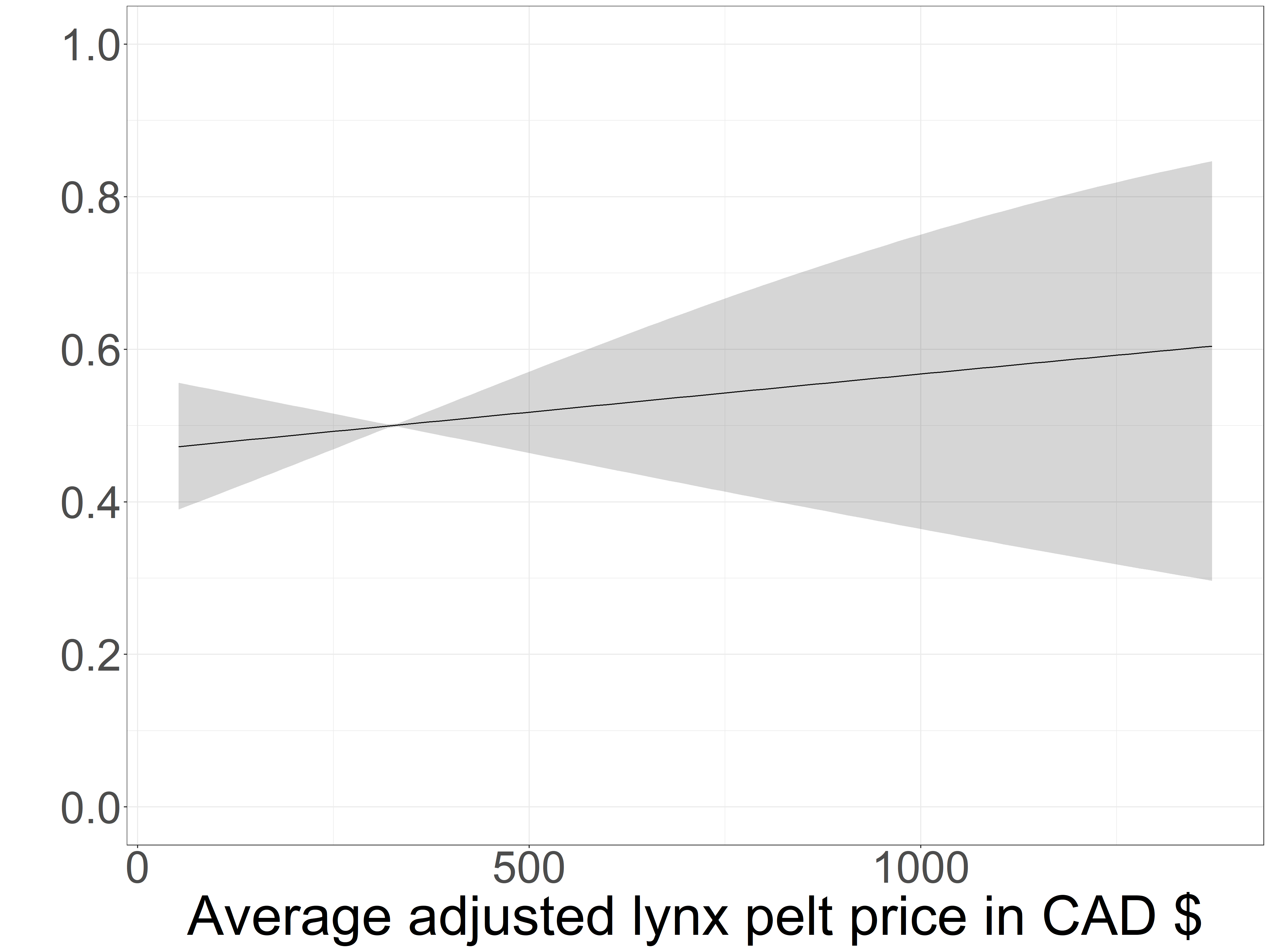


Figure S1. Effort covariates for predicting the probability of harvesting a Canada lynx between 1948 and 2017 across sampling units south of the boreal forest in Ontario, Canada.


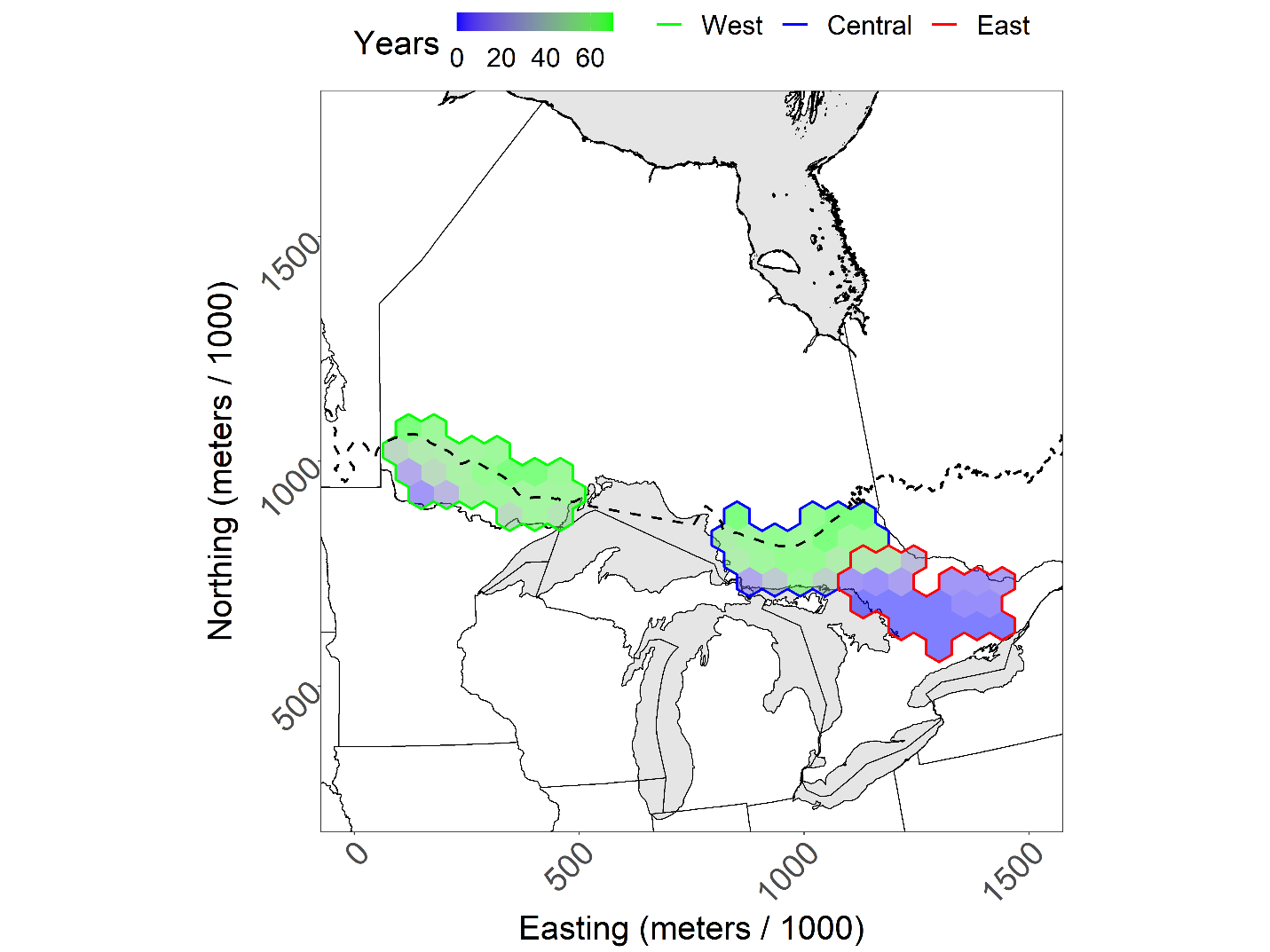


Figure S2. Frequency or number of years that each sampling unit was part of the southern Canada lynx range between 1948-2017 south of the boreal forest in Ontario, Canada. The dashed black line is the boreal forest southern limit by Brandt (2009). Spatial layers for administrative boundaries were gathered from the Database of Global Administrative Areas (<https://gadm.org/>).


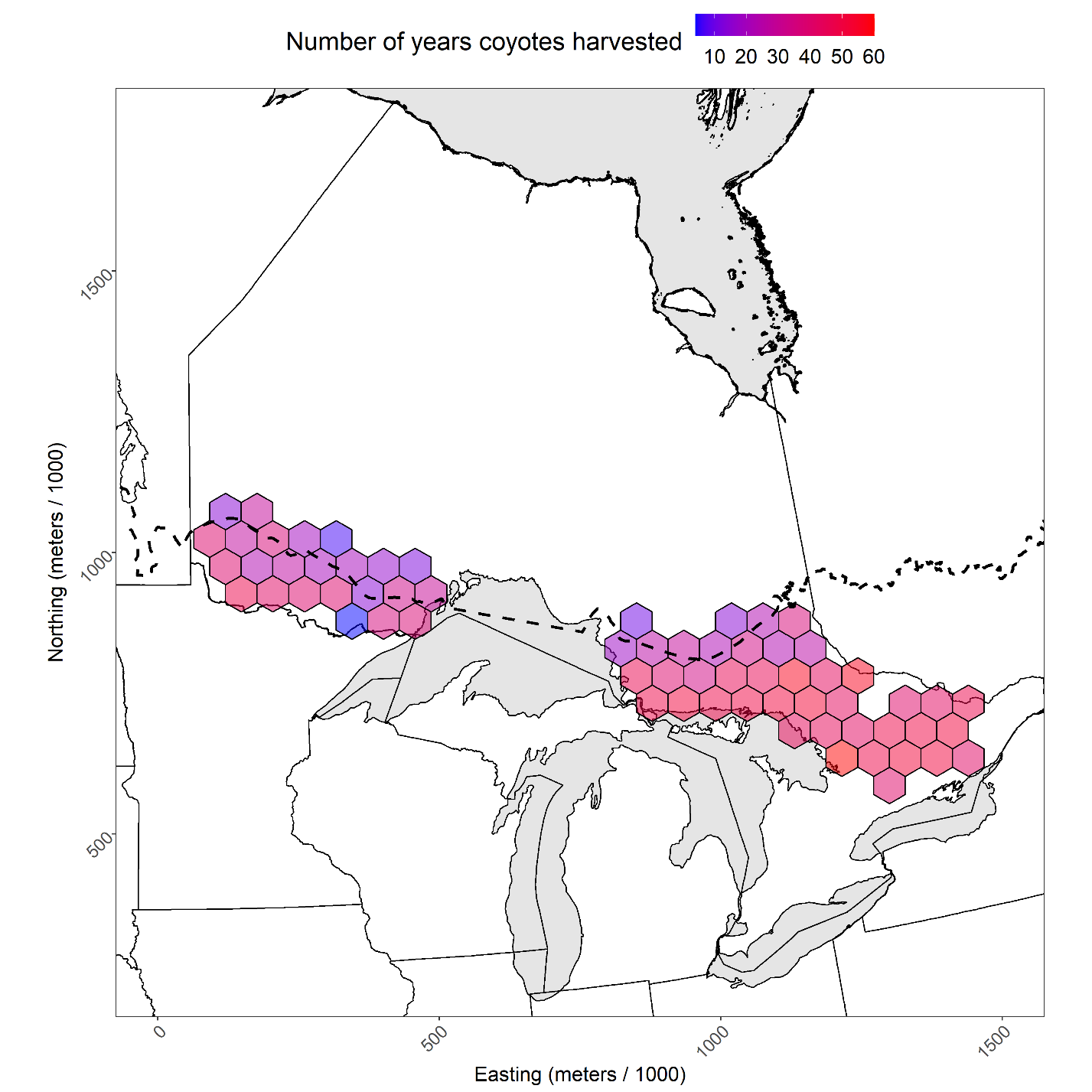


Figure S3. Number of years that coyotes were harvested within sampling units in the southern range of the Canada lynx in Ontario, Canada between 1948 and 2017. The dashed black line is the boreal forest southern limit by Brandt (2009). Spatial layers for administrative boundaries were gathered from the Database of Global Administrative Areas (https://gadm.org/).


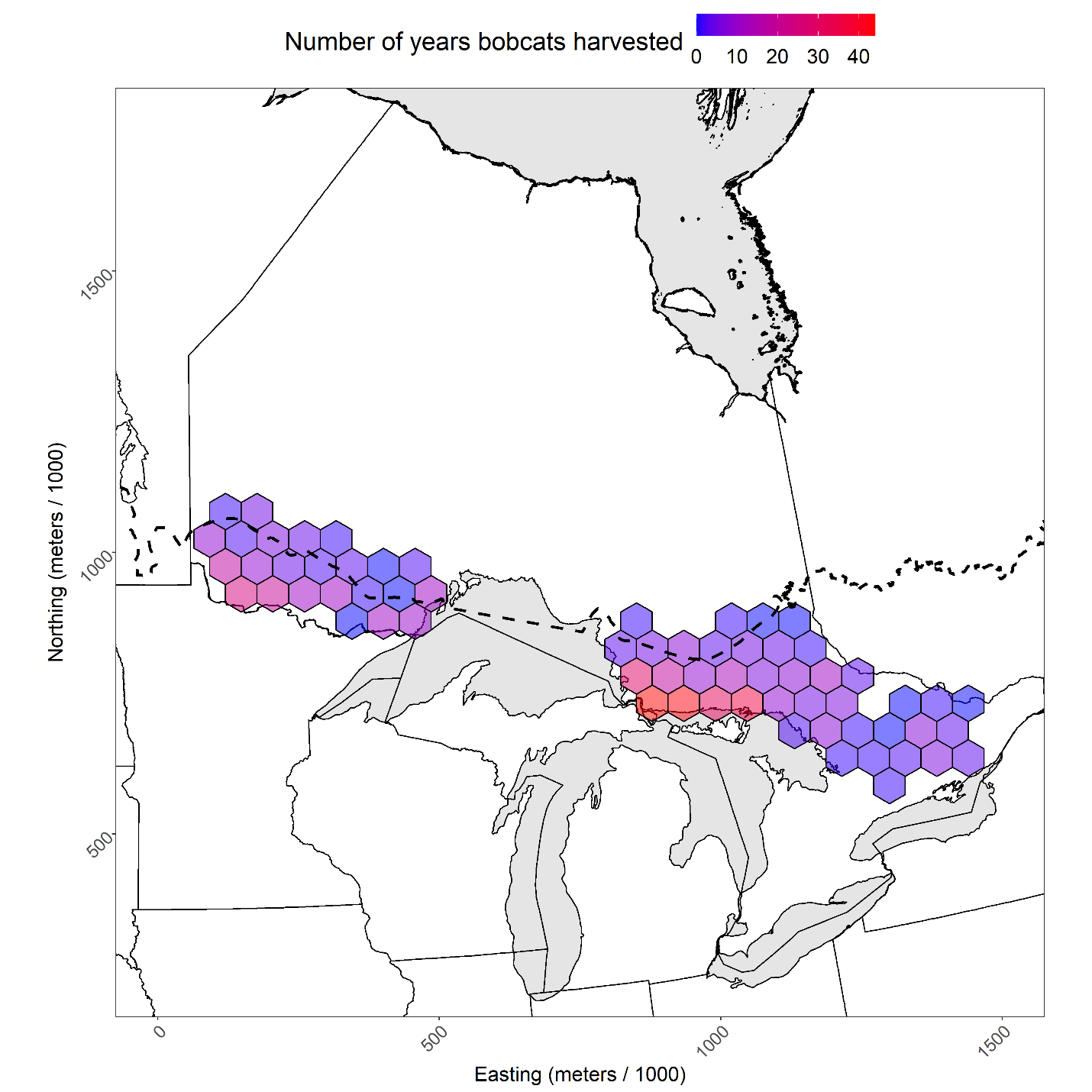


Figure S4. Number of years that bobcats were harvested within sampling units in the southern range of the Canada lynx in Ontario, Canada between 1948 and 2017. The dashed black line is the boreal forest southern limit by Brandt (2009). Spatial layers for administrative boundaries were gathered from the Database of Global Administrative Areas (https://gadm.org/).


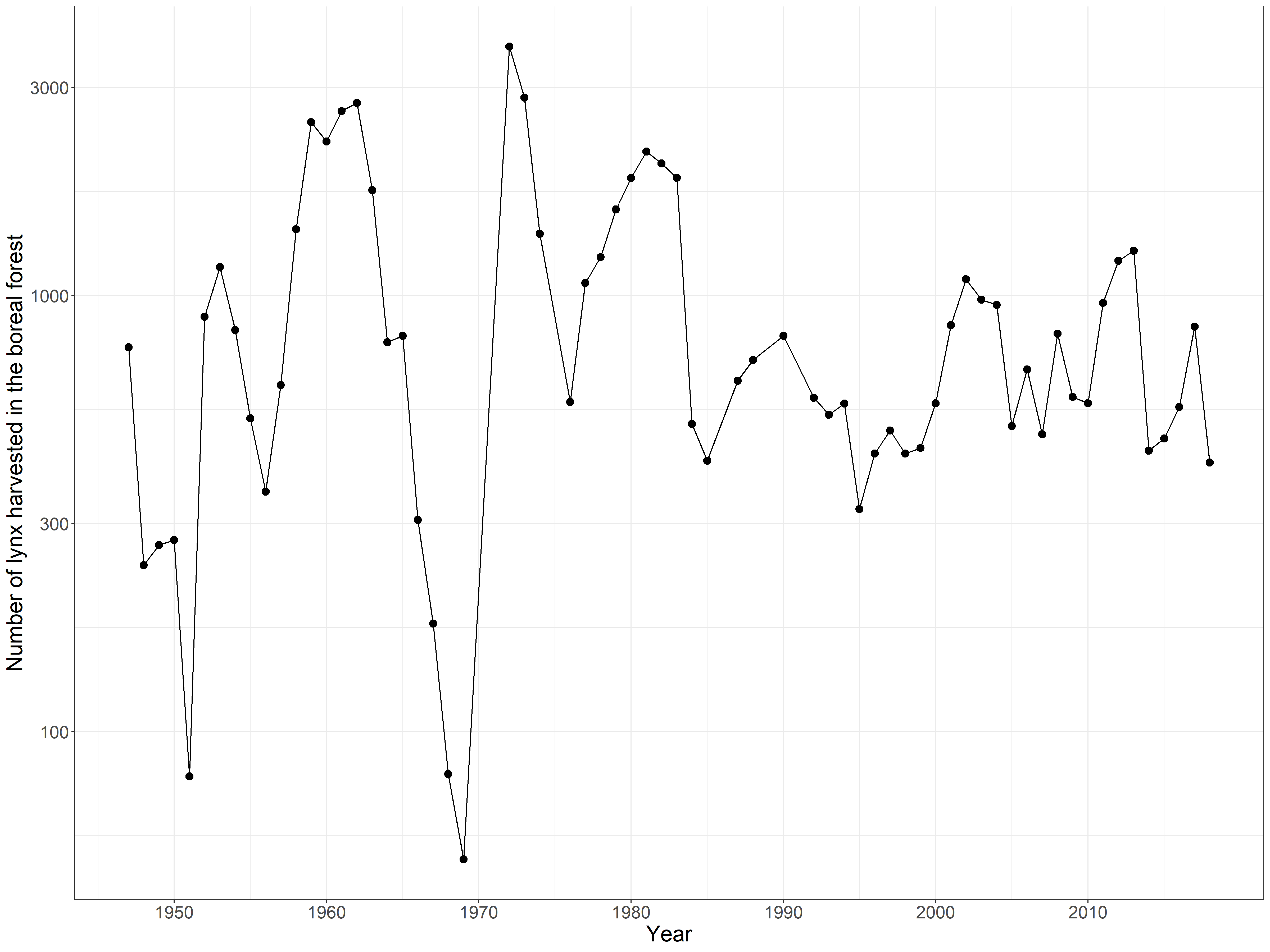


Figure S5. Number of Canada lynx harvested in the boreal forest of Ontario from 1948 to 2018. Records used to estimate the southern range at the southern boundary of the boreal forest were removed.
